# Evaluation of pro-apoptotic potential of taxifolin against liver cancer

**DOI:** 10.7717/peerj.11276

**Published:** 2021-05-25

**Authors:** Sania Safdar Butt, Khushbukhat Khan, Yasmin Badshah, Mehak Rafiq, Maria Shabbir

**Affiliations:** 1 Atta-Ur-Rahman School of Applied Biosciences, National University of Sciences and Technology, Islamabad, Pakistan; 2 Research Centre for Modelling and Simulation, National University of Sciences and Technology, Islamabad, Pakistan

**Keywords:** Liver Cancer, Pro-apoptotic, Taxifolin, VEGF, IC50, Hif1-α, Akt, Hepatocellular carcinoma cell line HepG2

## Abstract

Liver cancer is the second most common cause of cancer-induced deaths worldwide. Liver cirrhosis and cancer are a consequence of the abnormal angio-architecture formation of liver and formation of new blood vessels. This angiogenesis is driven by overexpression of hypoxia-inducible factor 1-alpha (Hif1-*α*) and vascular endothelial growth factor (VEGF). Apart from this, protein kinase B (Akt) is also impaired in liver cancer. Despite the advancement in conventional treatments, liver cancer remains largely incurable. Nowadays, the use of naturally occurring anticancer agents particularly flavonoids is subject to more attention due to their enhanced physicochemical properties. Therefore, this study underlines the use of a natural anticancer agent taxifolin in the treatment of liver cancer using hepatocellular carcinoma cell line HepG2 and Huh7. The aim of our study is to devise a natural and efficient solution for the disease prevalent in Pakistan. The study involved the assessment of binding of ligand taxifolin using molecular docking. The binding of taxifolin with the proteins (Hif1-*α*, VEGF and Akt) was calculated by docking using Vina and Chimera. Further evaluation was performed by cell viability assay (MTT 3-(4,5-dimethylthiazol-2-yl)-2,5-diphenyltetrazolium bromide (MTT) Assay), colony formation assay, cell migration assay, DNA ladder assay and flow cytometry. To see whether taxifolin directly affected expression levels, analysis of gene expression of Hif1-*α*, VEGF and Akt was performed using real-time polymerase chain reaction (qPCR) and western blotting. In silico docking experiments revealed that these proteins showed favorable docking scores with taxifolin. Treatment with taxifolin resulted in the inhibition of the liver cancer growth and migration, and induced apoptosis in HepG2 and Huh7 cell lines at an inhibitory concentration (IC50) value of 0.15 µM and 0.22 µM, respectively. The expression of HIF1-*α*, VEGF and Akt was significantly reduced in a dose- dependent manner. The inhibitory effect of taxifolin on hepatic cells suggested its chemopreventive and therapeutic potential. The studied compound taxifolin exhibited pronounced pro-apoptotic and hepatoprotective potential. Our study has confirmed the pro-apoptotic potential of taxifolin in liver cancer cell lines and will pave a way to the use of taxifolin as a chemotherapeutic agent after its further validation on the animal models and humans based epidemiological studies.

## Introduction

Liver cancer, like every other cancer, is manifested by the uncontrolled division and re-division and thus over-proliferation of the abnormal cells (Njei et al., 2015). Various risk factors that contribute to hepatocellular carcinoma (HCC) are: rise in the (non-alcoholic) fatty liver disease, alcohol and aflatoxin B1-exposed high prevalence of hepatitis B virus (HBV) and hepatitis C virus (HCV) infection and liver cirrhosis (Baffy, Brunt & Caldwell, 2012; El-Serag & Kanwal, 2014; Schütte et al., 2013). Epigenetic and genetic changes occur that includes the activation of oncogenes and inactivation of the tumor suppressor genes. Increase in the hepatocellular turnover, induced by inflammation, and DNA damage, induced by oxidative stress, leads to HCC disease manifestation (Farazi & DePinho, 2006).

Surveillance, Epidemiology and End Results data (SEER) has indicated that the cases of liver cancer are rising in parallel with 1.51 cases per 100,000 in 1973 and 6.20 (per 100,000) in 2011 (Schütte et al., 2015). HCC is the second most common cause of death in the world and ninth most common in the USA (Jemal et al., 2011).

During the event of liver injury the liver tissues are regenerated by epidermal growth factor (EGF), transforming growth factor (TGF), insulin-like growth factor (IGF) and VEGF (Höpfner, Schuppan & Scherübl, 2008). It has been previously investigated that in HCC, the platelet-derived growth factor receptor (PDGFR) and vascular endothelial growth factor receptor (VEGFR) signaling cascades and pathways such as Wnt-catenin, hedgehog, phosphoinositide 3-kinase (PI3K)/Akt/mTOR, IGF, tyrosine-protein kinase c-MET and mitogen-activated protein kinase (MAPK), show altered activity (Kudo, 2008).

For VEGFR, PDGFR, and EGFR, the common downstream pathway is the MAPK pathway. This pathway is involved in signal transduction downstream to a small GTPase protein Ras (rat sarcoma), integrin complexes, and cytokine receptors. The pathway is found to play a very important role in liver cancer as the growth and survival of HCC are due to the activation of MAPK (Llovet & Bruix, 2008).

Doxorubicin is one of the first line chemotherapeutic agents in treatment of HCC. The only approved drug targeted against liver cancer is Sorafenib (Swamy et al., 2017). Conventional treatment against liver cancer has many limitations, so this study employs the use of complementary and alternative medicine against liver cancer. In this study, we have used a natural flavonoid compound taxifolin (also known as dihydro-quercetin (DHQ)), found in onions, Douglas fir bark (Kiehlmann & Slade, 2003), French maritime bark (Rohdewald, 2002), and milk thistle (Wallace, Carrier & Clausen, 2005). Until now, taxifolin has been used in complex preparations such as pycnogenol, venoruton, and silymarin (Legalon™) and has a very rare use as a single compound. For instance, the extract from the milk thistle plant seeds (*Silybummarianum*) is employed for chronic liver disease treatment in Germany (Blumenthal, 1998) and also by the European Medicine Agency in the recipients of liver transplant for preventing the recurrence of hepatitis C (Weidmann, 2012). Silymarin is a licensed hepatoprotective drug employed in the supplementary treatment of liver cirrhosis and inflammatory liver disease as well as for toxic liver damage treatment. The compound also exhibits the anticancer property by modulating antioxidant responses, Wnt and immunomodulatory pathways ultimately causing the activation of anti-oxidant redox element (ARE) and, by this regulation mechanism, acts as a potential chemopreventive agent (Manigandan et al., 2015). In liver carcinoma, taxifolin and other silymarin extracts exert beneficial effects by attenuation of the mast cells recruitment that further inhibit angiogenesis and invasion by decreasing the expression of matrix metalloproteinases MMP-9 and MMP-2 (Ramakrishnan et al., 2009). In this study, we have investigated the anti-angiogenic and pro-apoptotic potential of taxifolin against liver cancer and the subsequent effects of taxifolin dose on Hif1-*α*,VEGF and Akt expression.

## Materials & Methods

### Molecular docking and bioinformatics analysis

Auto Dock Vina (Trott & Olson, 2010) and Chimera (Version 1.13) was used to perform Docking of the compound Taxifolin (PubChem ID 439533) against the proteins Akt (PDB ID: 1GZO) , VEGF (PDB ID: 3QTK) and HIF-1*α* (PDB ID: 4H6j). Docking was performed through Vina using Opal services in Chimera 1.13 by selecting the appropriate options. The hydrogen bonds between the receptor and ligand were visualized and docking scores were retrieved.

### Cell culture and treatment

Human hepatocellular carcinoma cells HepG2 and HuH7 and kidney epithelial cells Vero were obtained from National Control Laboratory for Biologicals (National Institute of Health Islamabad). All cell lines were grown in DMEM (Gibco by Life Technologies) supplemented with 10% Fetal Bovine serum (FBS) (Gibco by Life Technologies) and 1% penicillin-streptomycin with 5% CO_2_ at 37 °C. Taxifolin (Sigma Aldrich) dissolved in Dimethyl sulfoxide (0.1% (v/v)) was employed for the treatment of HepG2 and Huh7 cells at a confluency of ∼70%. Cells were treated with taxifolin at 0.1 µM, 0.125 µM, 0.15 µM, 0.20 µM and 0.22 µM for 24 h.

### Cell viability assay

MTT Assay was used to study the effect of Taxifolin on the viability of HepG2, HuH7 and Vero cell lines. The cells were plated (1 × 104 cells per well) in one mL of complete cell culture medium, containing 0.1 µM, 0.125 µM, and 0.15 µM concentrations of taxifolin in 96 well plates. Cells were incubated at room temperature for 24 h. After incubation, 100 µL of MTT (5 mg/ml:1XPBS) was added to each well and incubated for 2–3 h in humidified atmosphere. DMSO (100 µL) was then added as solubilization solution and again plate was put overnight in humidified incubator. Afterwards, cell culture plates were centrifuged (1,800 × g for 5 min at 4 °C).The absorbance of 550 nM was recorded on the microplate reader. DMSO treated cells were taken as 100% control and the effect of taxifolin on the inhibition of cell growth was calculated as the % cell viability.

### Colony formation assay

The colony formation assay (clonogenic assay) was performed in order to assess the effect of treatment on clonogenic survival of liver cancer cells. HepG2 and HuH7 cells were treated with increasing concentrations (0.1 µM, 0.12 µM, and 0.15 µM) of taxifolin in DMEM medium. Following treatment, the cells were re-plated in triplicates on a 12-well tissue culture plate with 3000 cells/well and cultured in 5% CO_2_ at 37 °C for 14 days with the growth media being replaced every four days. The cells were stained with 0.5% crystal violet (in methanol: H_2_O; 1:1) and the pictures were taken using a digital camera.

### Cell migration assay

HepG2 and HuH7 cells were seeded in 6-well cell culture plates and were allowed to spread, attach and form a monolayer for 24 h. After attachment, the cells were scratched with a sterile pipette tip and then incubated. At 80% confluence they were given a treatment of 0.125 µM and 0.15 µM to examine the effect of taxifolin on the migration of HCC. After the next 24 h, the images of the cell movement were captured at regular intervals through LSM 410 microscope (Zeiss, Germany).

### RNA extraction and real-time PCR

Total RNA was isolated from the cells by using trizol solution (Wizbio) according to the manufacturer’s instructions. The concentration of RNA was measured spectrophotometrically and cDNA was synthesized, following the manufacturer protocol (wiz Script cDNA synthesis kit). The reaction mixture for qPCR was prepared using 10 µL of Wiz Pure qPCR master mix (SYBR), 6 µM of reverse and forward primers, and 10 µg cDNA with RNAase free water added to a total volume of 20 µL. The amplification was performed for 40 cycles with the following factors: 95 °C incubation for 10 minutes, then 95 °C for 15 s, amplification at 61 °C for 1 min and real time analysis at 75 °C for 45 s. The data was collected at 61 °C and 7,300 system SDS software was used for the analysis of the data. For normalization, beta-actin primers were used. 2-ΔΔCT method was employed for the gene expression quantification. The primer sequence used were Hif1-*α* Forward: 5′ CAGATCTCGGCGAAGTAAAG 3′, Hif1-*α* Reverse 5′ TCACAGAGGCCTTATCAAGAT G 3′; VEGF Forward: 5′ CGAGGGCCTGGAGTGTG 3′, VEGF Reverse: 5′ CCGCATAATCTGCATGGTGAT 3′; Akt Forward 5′ TTCTGCAGCTATGCGCAATGTG 3′, Akt Reverse: 5′ TGGCCAGCATACCATAGTGAGGTT 3′.

### Protein extraction and Western blot analysis

Protein extraction was performed after the treatment of taxifolin in Huh7 and HepG2 cells. Procedure included the addition of ice-cold lysis buffer (consisting of one mmol/l EDTA, one mmol/l EGTA, 150 mmol/l NaCl, 50 nmol/l Tris–HCl, 20 mmol/l NaF, 1% Triton X-100, 0.5% Nonidet P-40, one mmol/l PMSF and 100 mmol/l Na_3_VO_4_ with pH 7.4), along with protease inhibitors (Calbiochem, Germany), to the cells and subjected to incubation for 20 min at 0 °C. For immunoblotting, 8–12% poly acrylamide gels were prepared for resolving proteins. Resolved proteins were transferred to nitrocellulose membrane and probed with monoclonal primary antibodies. After incubation with secondary antibodies, proteins of interest were detected through chemiluminescence autoradiography.

### DNA ladder assay

DNA was isolated using phenol-chloroform method to estimate the DNA damage. 5 µg of DNA was loaded in 1.5% agarose gel that contained 1.0 µg/ml ethidium bromide (EtBr) including the DNA standards 0.5 µg per well. Electrophoresis was performed for 45 min at 100 volts. The gel was studied under the gel doc system and using digital camera was photographed after observing.

### Flow cytometry

Taxifolin-treated HepG2 and Huh7 cells were trypsinized and fixed in Paraformaldehyde:1XPBS (1%) for 60 min. Then, cells were washed with cold PBS twice and centrifuged. Ice chilled 70% ethanol was used to suspend and store over-night the resultant pellet. The cells were again centrifuged (1000 rpm for 5 min) and washing with cold PBS was done to remove ethanol from obtained pellet. Propidium iodide and FITC were used to label the cells through Apo-Direct Kit (BD Pharmagen, CA) as instructed by manufacturer and analyzed through FACScan (Becton Dickinson, NJ). Approximately 10,000 cells per sample were picked and ModFitLT software (Verily Software House, ME) was used to analyze DNA histograms.

### Statistical analysis

Statistical analysis was performed using one way ANOVA (multiple comparisons) in graph pad prism and *p* value less than 0.05 was considered statistically significant.

**Table 1:** Docking score and RMSD values of Hif1-*α*, VEGF and Akt protein docked with ligand taxifolin.

**Name of protein**	**Diagram**	**Docking score**	**RMSD upper bond**	**RMSD lower bond**	**H bonds all**	**H bonds ligand atoms**	**H bonds receptor atoms**
Hif1-*α*	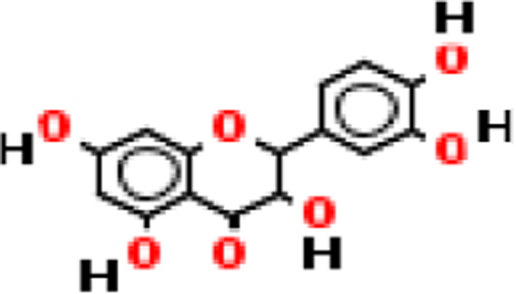	11.6	0.0	0.0	3	3	3
		14.6	1.595	7.169	5	4	4
VEGF	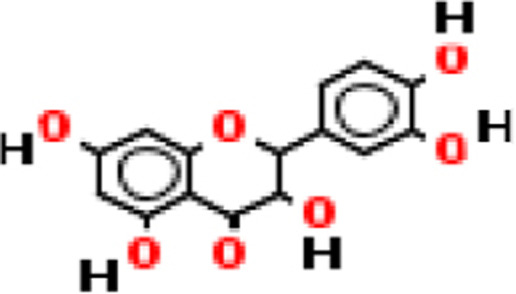	−9.4	0.0	0.0	3	3	2
		−7.9	1.953	6.886	3	2	3
		−7.8	2.219	4.118	2	2	2
Akt	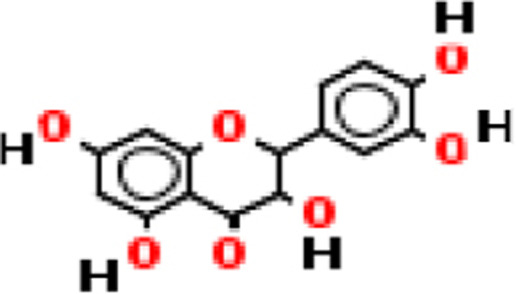	−7.9	0.613	1.429	1	1	1
		−7.2	1.915	2.327	1	1	1
		−7.7	1.497	6.507	1	1	1

## Results

### Binding interactions of taxifolin with the binding domains of VEGF, Akt and Hif1-*α*

**Figure 1:**
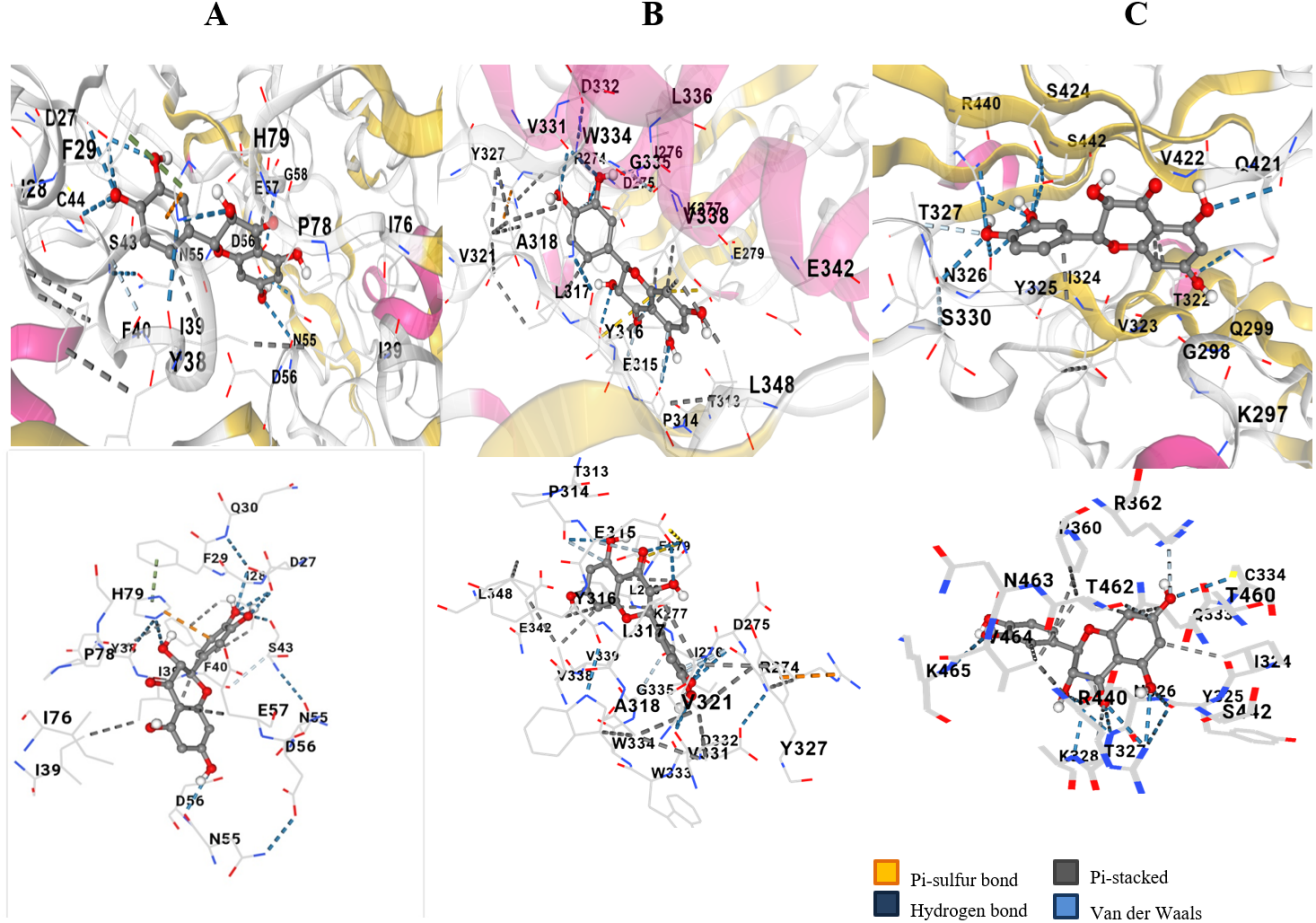
In silico docking of taxifolin with VEGF. (A) Akt (B) and Hif1*α* (C). Taxifolin interacts with numerous amino acids in binding pocket of VEGF, Akt and Hif1*α* through the –OH groups and forms hydrogen bonds. Atoms highlighted in red and white in taxifolin structure depict hydroxyl groups and that interaction is highlighted with a blue dotted line.

The hydroxyl group and the oxygen atom on taxifolin resulted in binding specifically with the Cysteine 44 position of VEGF (Fig. 1A). Docking resulted in ten different scores through which the top three hits having maximum number of hydrogen bonds were selected. The score of −9.4, −7.9 and −7.8 was selected. Two hydrogen bonds and high negative docking score depicted that VEGF had the most favorable docking with ligand taxifolin. Akt showed binding affinity to the ligand Taxifolin. This was observed that the hydroxyl group of the ligand showed interaction with the phenylalanine residue of Akt protein (Fig. 1B). Ten possible scores of docking were obtained and the top three hits having maximum hydrogen bonds were selected. The hydroxyl group on the ligand Taxifolin was found to interact with Hif1-*α* at the TYR325 (tyrosine) by means of hydrogen bond. The ligand docked with the Hif1-*α* protein with two possible orientations depicting the docking score of 11.6 (RMSD l.b and u.b 0.0) and 14.6 (1.5 and 7.16) respectively. These results showed a favorable binding interaction between our ligand and the protein Hif1-*α* (Fig. 1C). Table 1 depicts the docking score, RMSD values and the hydrogen bonds that result in the ligand protein interactions values. VEGF, Akt and Hif1-*α* were shown to exhibit docking scores viz., −9.4, −7.9 and 11.6 respectively.

### Inhibitory effect of Taxifolin ongrowth and viability of hepatic carcinoma cell lines

**Figure 2:**
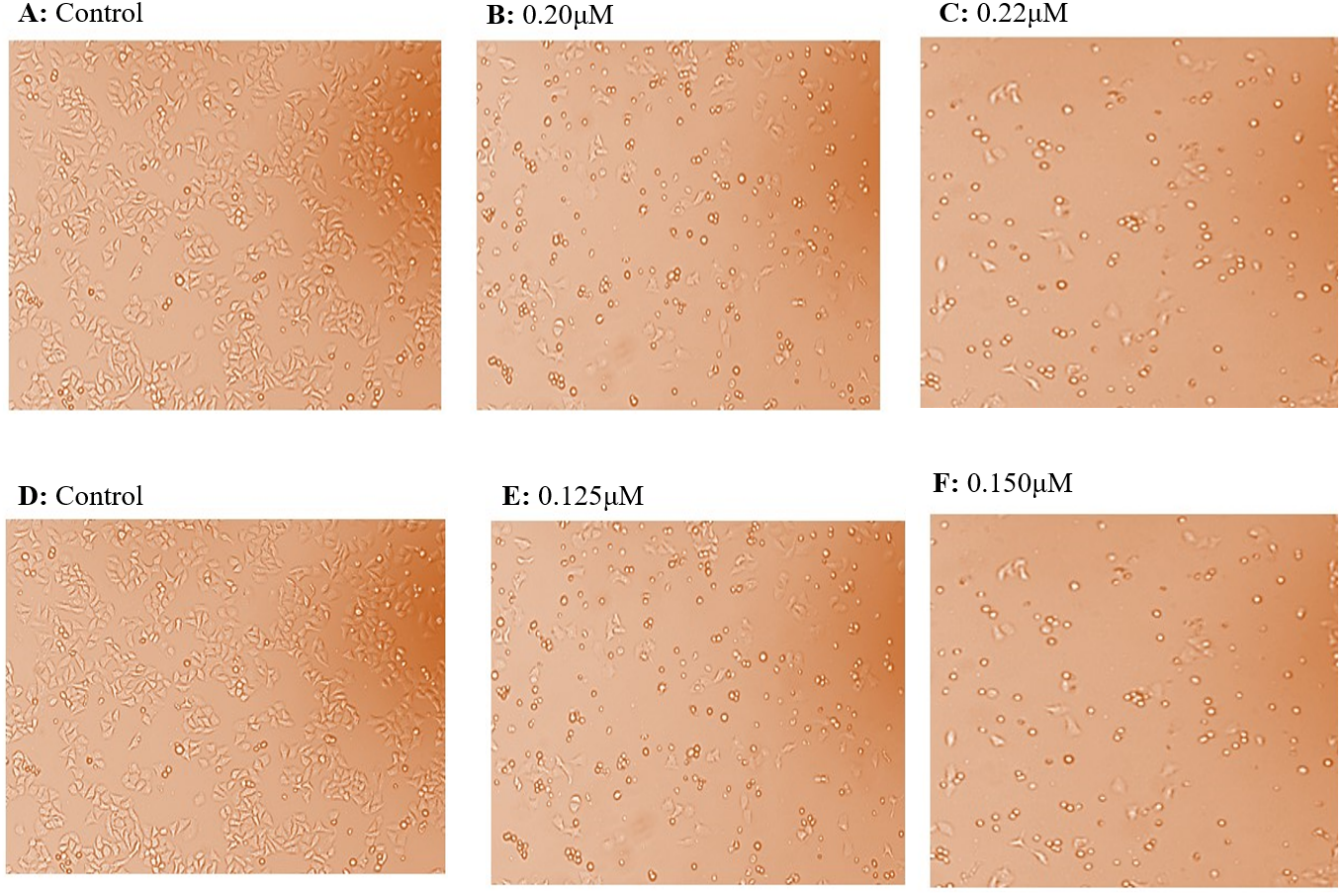
Effect of taxifolin treatment on growth of hepatic cancer cells (Huh7 and HepG2 Cells) with different concentrations. Cell growth and treatment of Huh7 and HepG2 Cells with different concentrations of taxifolin. (A) Huh7 control cell line. (B) Taxifolin treatment at 0.20 µM. (C) Taxifolin treatment at 0.220 µM. (D) HepG2 control cell line. (E) Taxifolin treatment at 0.125 µM. (F) Taxifolin treatment at 0.150 µM. Cell growth in both cell lines decreased in dose-dependent manner.

HepG2 and Huh7 cell lines were grown and maintained in DMEM media. The normal kidney cells (Vero cells) were also grown in DMEM media and were seeded continuously for cytotoxicity experiments. HepG2 cells were given the treatment with taxifolin at different concentrations of 0.10 µM, 0.125 µM, and 0.150 µM (Fig. 2). The IC50 for HepG2 was achieved at a concentration of 0.15 µM, where 50% of cancer cells growth was inhibited. Huh7 cells were treated with taxifolin at different concentrations of 0.200 µM and 0.220 µM. The IC50 of taxifolin for Huh7 cells was 0.220 µM. Cell counting was done by trypan blue assay, performed before each cell culture experiment.

**Figure 3:**
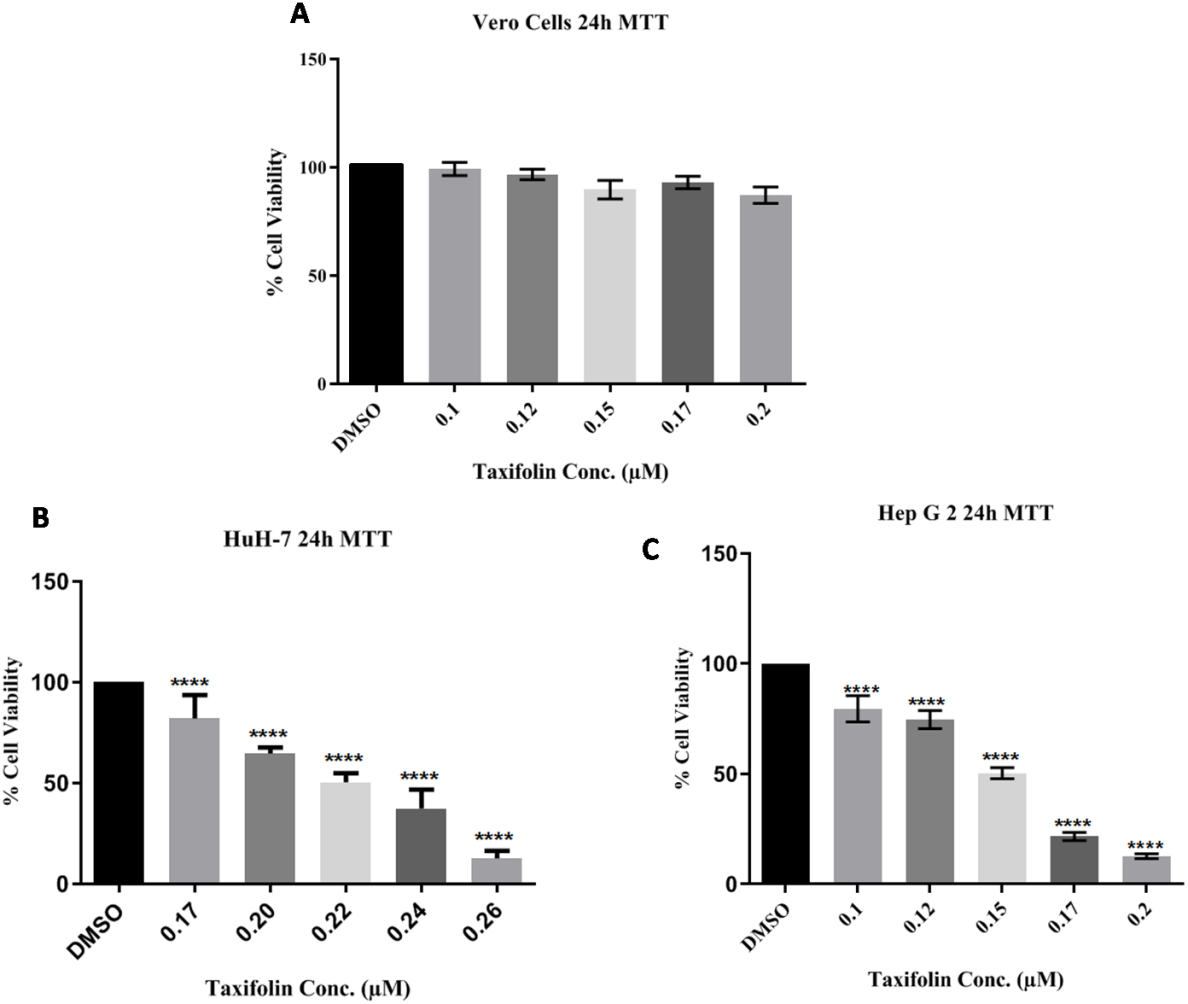
Taxifolin inhibits growth and reduce viability of hepatic cancer cells. Vero (A), Huh7 (B) and HepG2 cells (C) were treated with taxifolin for 24 h and MTT assay was performed to assess cell viability. Taxifolin did not influenced viability of non-carcinogenic epithelial Vero cells. However, its varying doses inhibited growth of Huh7 and HepG2 cells. **** *p* < 0.01 was considered statistically significant.

In three independent experiments, cell cytotoxicitywas measured as a percentage of corresponding control (value of untreated cells). The toxicity for taxifolin was estimated on Vero cells; MTT assay for Vero was performed with respect to different taxifolin concentrations i.e., 0.1 µM, 0.125 µM, 0.15 µM, 0.17 µM and 0.20 µM for 24 h. A distinctive pattern for IC50 values at the three tested cell lines Vero, Huh7 and HepG2 was observed. Treatment of Vero cell lines with taxifolin did not pose any cytotoxic effect (Fig. 3A).

Since taxifolin is known to exhibit anti-cancer activities, it was investigated whether taxifolin extract would be more efficacious in the inhibition of growth and viability of liver cancer cells. We performed MTT assay against liver cancer cells (Huh7 and HepG2) for the investigation of the anti-proliferative potential of taxifolin and checked the percentage of cancer cells relative to the control cells. It was observed that taxifolin treatment (0.1 µM, 0.125 µM and 0.15 µM, 0.17 µM and 0.20 µM for 24 h) to liver cancer cells caused cell growth inhibition in a dose-dependent manner revealing that both cell lines responded to taxifolin treatment within 24 h. As depicted in Figs. 3B and 3C, the IC50 value of taxifolin treated Huh7 and HepG2 cells was 0.22 µM and 0.150 µM (24 h), respectively. At half maximal inhibitory concentration (IC50) it was observed that the growth of cancer cells was inhibited by 50%. In viable cells, mitochondrial dehydrogenase enzyme resulted in the cellular reduction of MTT; soluble yellow tetrazolium salt to purple colored formazan product formation. Significant decrease in viability of HepG2 was observed in a dose-dependent manner. Each experiment was performed in triplicates and the results of three independent experiments are reported. The percent cell viability of the HepG2 control cells (control; DMSO) was 100%, with a considerable reduction of 25% in the cells treated with a dose of 0.125 µM and 50% in cells at 0.15 µM. Similar trend was observed in Huh7 cells after treatment with varying concentrations of taxifolin, indicating the decline in viability of liver cancer cells upon increasing taxifolin dosage.

### Reduction of clonogenic survival and migratory potential of hepatic cancercells after taxifolin treatment

**Figure 4:**
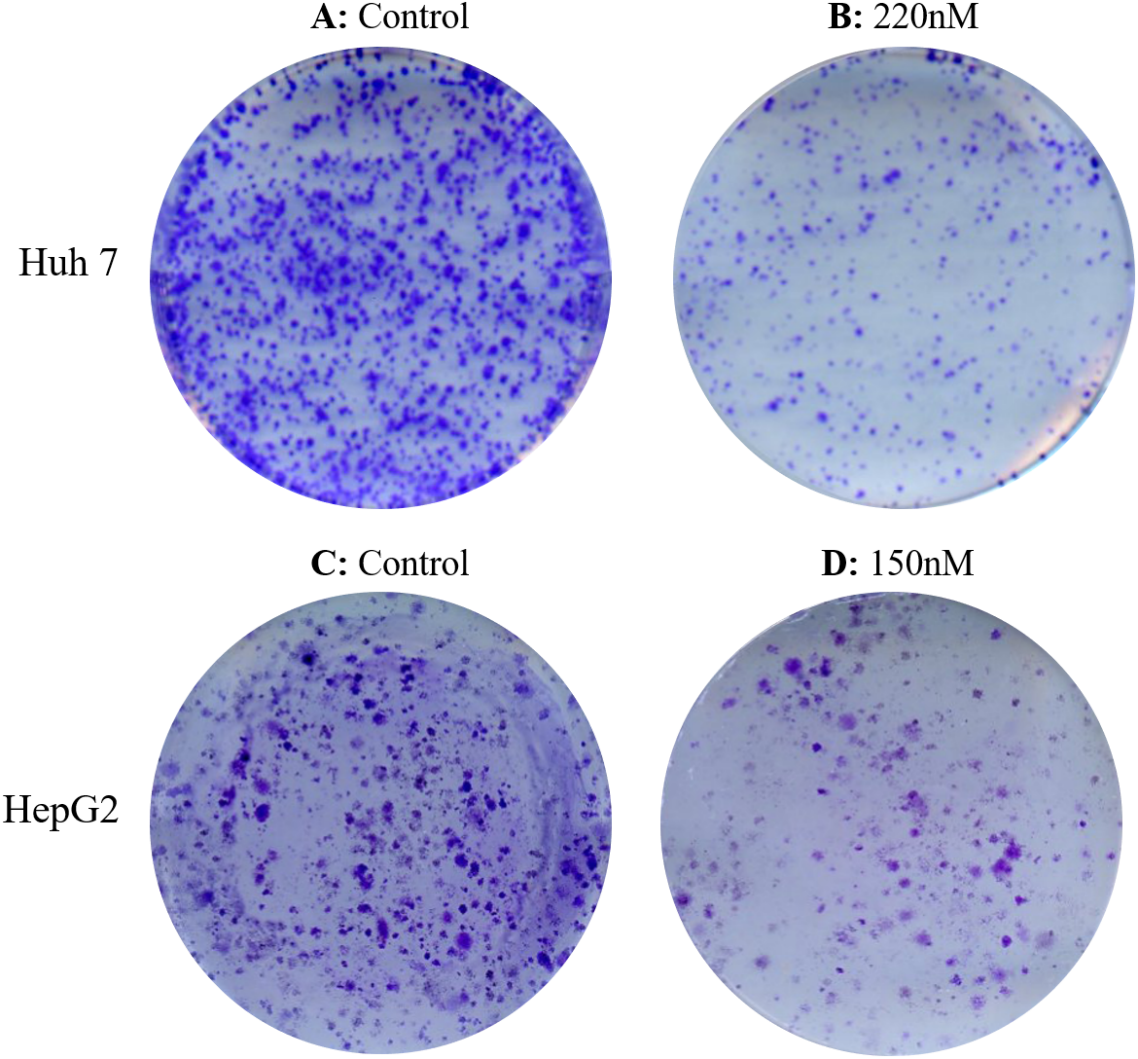
Detection of dose dependent effect of taxifolin on clonogenicity of hepatic cancer cells. Colonogenecity in taxifolin treated Huh7 (A-B) and HepG2 (C-D) cells. Taxifolin treatment for 24 h remarkably reduced the colony forming potential of hepatic carcinoma cells.

Both cell lines were treated with increasing concentrations of taxifolin (0.1 µM, 0.125 µM, and 0.15 µM). After incubation and treatment of HepG2 and Huh7 cells for 14 days, treated cells exhibited a dose-dependent inhibition in colony formation relative to controls (untreated). Treatment of cell lines with increasing concentration of taxifolin resulted in a decline in cell survival and their reproductive ability to form clone (a large colony), with respect to the control. Figure 4 shows affected clonogenicity of Huh7 and HepG2 cells upon taxifolin treatment in comparison to control. Clonogenicity of liver cancer cells was highest in control. The surviving fraction declined as the concentration was increased to 0.1 µM and even further at 0.125 µM. Finally at the concentration of 0.150 µM for HepG2 and 0.220 µM for Huh7, taxifolin minimized the surviving fraction affirming its cytotoxic role for liver cancer cells (Clonogenicity 0.1 µM > 0.125 µM > 0.150 µM).

**Figure 5:**
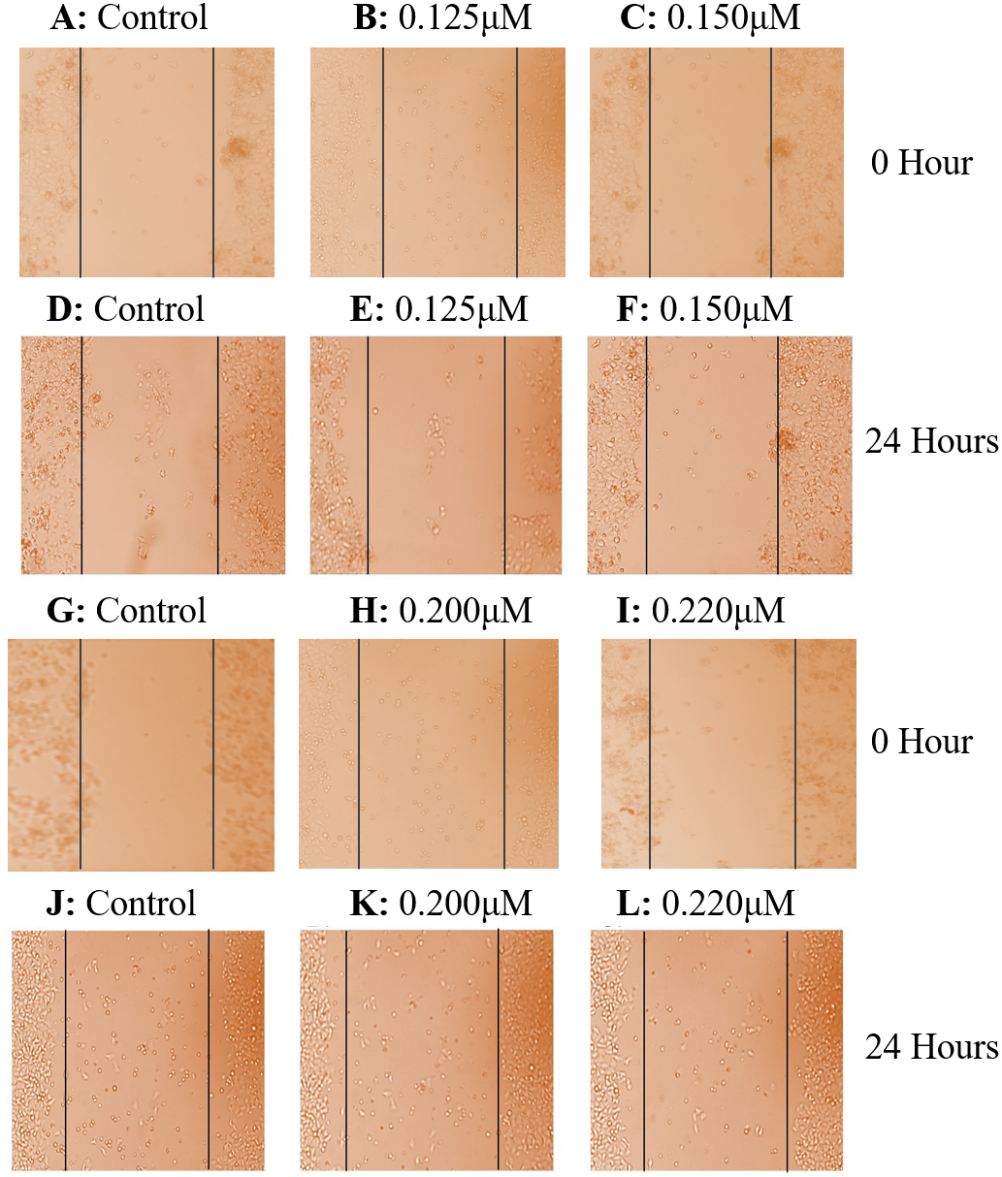
Effect of taxifolin on cell migration in hepatocellular carcinoma. Effect of taxifolin on migratory potential of hepatic cancer Huh7 and HepG2 cell lines. (A–F) Scratch assay for HepG2 cell where Migration inhibition by taxifolin was assessed at 0.125 µM and 0.150 µM. (G–L) Scratch assay for Huh7 cells where migration inhibition by taxifolin was assessed at 0.20 µM and 0.220 µM. A-D depict cells at hour 0 and D-F depict cells after 24 h. Taxifolin induced dose-dependent inhibition of migration in both cell lines.

Cell scratch assay exhibited that after culturing for 24 h, taxifolin significantly suppressed the migration of HepG2 and Huh7cells. Control cells exhibited a significant potential to migrate having no hindrance in cell motility (Figs. 5A and 5D). In both treated cell lines, taxifolin caused inhibition in the migration of liver cancer cells and reduced the speed of wound healing in a concentration-dependent manner. The results indicated that treatment of HepG2 cells with dose 0.125 µM and 0.15 µM and Huh7 cells with dose 0.20 µM and 0.22 µM slowed the motility and the wound remained unfilled at 24 h. The migration distance decreased significantly upon increasing concentration of taxifolin which depicts that taxifolin reduced the migratory capability of hepatic cells in vitro. The migration ability of HepG2 cells at 0.125 µM concentration of taxifolin was notably decreased (Fig. 5B) whereas further minimized at 0.15 µM (Fig. 5C). Similarly, taxifolin treatment also brought about significant reduction in migration potential of Huh7 cells at 0.20 µM (Fig. 5E) and its increased concentration (0.22 µM) further decreased their migration (Fig. 5F).

### Taxifolin induces DNA damage as well as apoptosis in hepatic cancer cells

**Figure 6:**
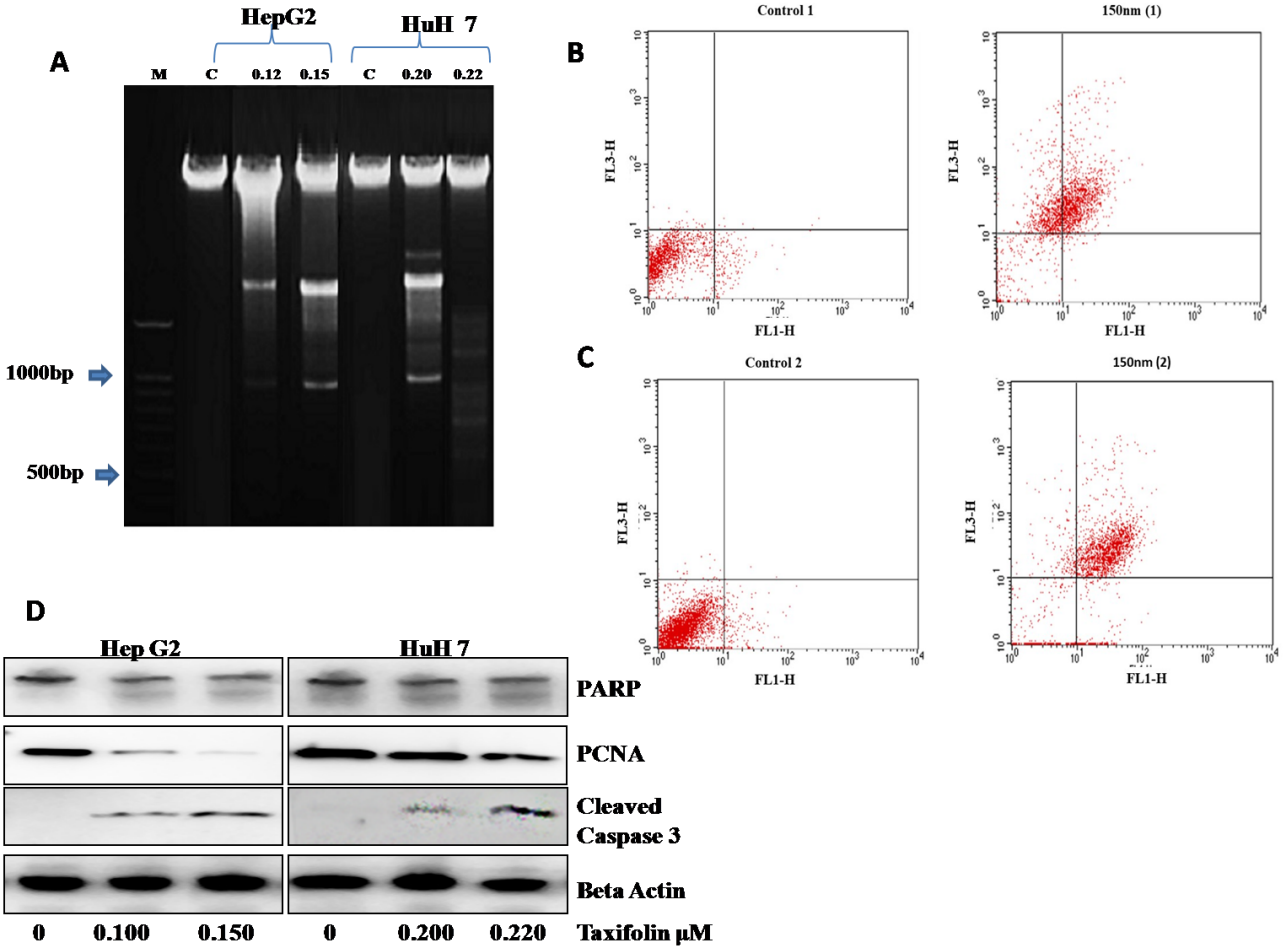
Taxifolin induce apoptosis in hepatic cancer cells. (A) DNA fragmentation visualized on 1% agrose gel. M represents lane with DNA ladder and C refers to control. DNA breakage is more prominent in 0.15 µM taxifolin- treated HepG2 cells and 0.22 µM taxifolin-treated Huh7 cells. (B & C) Flow cytometry analysis of annexin V- (FL1-H) and PI- (FL3-H) labeled cells. Huh7 (B) and HepG2 cells (C) were treated with 0.220 µM and 0.150 µM of taxifolin for 24 h. Control 1/2 are the untreated cells while 150 nM (1/2) are Huh7 and HepG2 cells treated with taxifolin. These cells were taken as a replicate for the validation of results. In the given dot plot, FL1 = Annexin V-FITC and FL3 = PI) (Real Time PCR) experiment performed in triplicate (mean ± S.D), *p* < 0.029.

Taxifolin influence on DNA damage and apoptosis was also assessed. DNA ladder assay showed that DNA damage was present in control as well as the DMSO treated group. Gel electrophoresis was used to examine DNA fragmentation. Control HepG2 cells did not present any DNA fragmentation signifying a high molecular weight DNA (intact DNA) (Fig. 6A). In contrast, the cells treated with taxifolin at 0.125 µM and 0.15 µM for 24 h resulted in fragmented bands (DNA breakage) forming a ladder pattern. This laddering of DNA depicts that DNA damage is a characteristic feature of apoptosis. The DNA fragmentation pattern in the cells treated at 0.125 µM was significantly less prominent than the cells treated at 0.15 µM concentration of taxifolin.

Next, the number of cells going through apoptosis after taxifolin treatment was assessed through flow cytometric analysis. A significant increase in the apoptotic population of cells in both cell lines was observed. In Figs. 6B and 6C, *x*-axis (FL1-H) refers to cells labeled with Annexin-V while *y*-axis refers to the cells labeled with propidium iodide (PI). PI-positive cells have heavily injured plasma membrane and are probably dead (shown in upper left quadrant). Annexin-V-positive cells represent cell population in initial phase of apoptosis (shown in lower right quadrant). Cells in late apoptotic phase binds both PI and Annexin-V (shown in upper right quadrant) while healthy cells bind with neither (shown in lower left quadrant). Dose-dependent increase in the ratio of dead cells to live cells was also recorded (Table S1), suggesting a positive correlation between taxifolin concentration and induction of cellular apoptosis.

Taxifolin influence on the expression of apoptotic markers (caspase-3, PCNA and PARP) was also assessed through western blotting (Fig. 6D). Immunoblot analysis revealed that PCNA expression in taxifolin-treated Huh7 and HepG2 cells was remarkably reduced while taxifolin induced PARP cleavage and caspase-3 activation was also seen. Furthermore, taxifolin pro-apoptotic influence was concentration-dependent and maximum reduction in protein levels of truncated-PARP and PCNA and increased activated caspase-3 was observed at 0.150 µM in HepG2 and 0.220 µM in Huh7 cells.

### Taxifolin causes downregulation of Hif1-*α*, VEGF and Aktexpression in hepatic cancer cells

**Figure 7:**
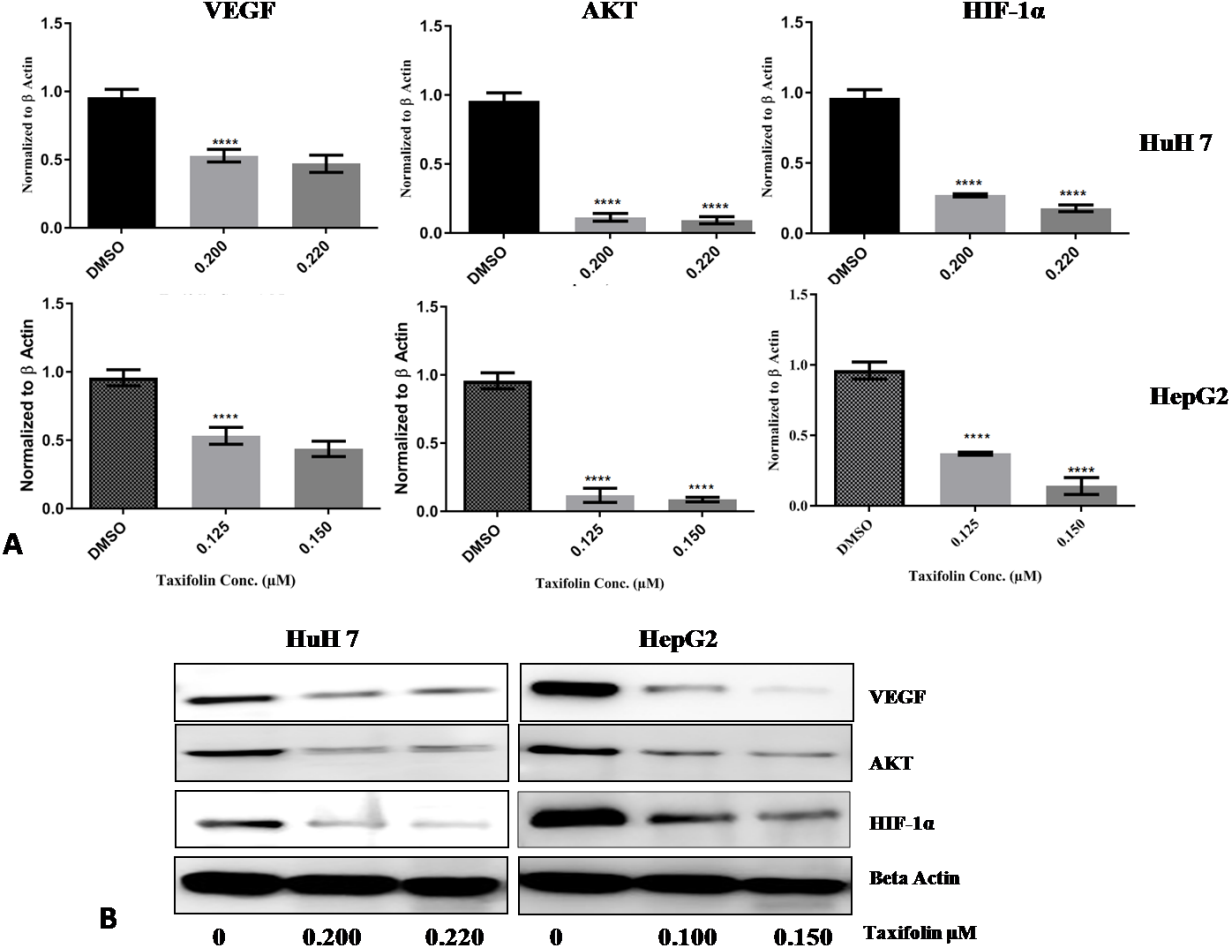
Taxifolin down regulates the mRNA and protein expression of VEGF, AKT and Hif1*α*. (A) qRT-PCR analysis revealed that taxifolin treatment for 2 h significantly downregulated the expression of VEGF, AKT and Hif1*α* in dose-dependent manner. The data expressed as fold change represent the mean ±  standard errors experiments performed in triplicates where **** *P* < 0.05 represents significance of data. (B) Taxifolin influence on VEGF, AKT and Hif1*α* protein expression is affirmed through immunoblotting analysis that also depicted remarkable attenuation in protein concentrations of these proteins after taxifolin treatment. The immunoblots shown are representative of three independent experiments with similar results.

Taxifolin influence on transcription and translation of Hif1- *α*, VEGF and Akt in HepG2 and Huh7 cells was determined by qPCR and immunoblotting. Both hepatic carcinoma cell lines were treated with taxifolin for 24 h and then assessed for taxifolin effect on Hif1-*α*, VEGF and Akt mRNA and protein expression level. mRNA expression of Hif1-*α* was significantly decreased (*p* < 0.001) in taxifolin treated cells in comparison to control cells. Figure 7A shows the mean fold change normalized to *β*-actin plotted against the two concentrations of taxifolin. Taxifolin resulted in a 0.6 fold decrease in Hif1-*α* at 0.125 µM and 1 fold decrease at 0.15 µM concentration. These results confirmed that taxifolin is an effective inhibitor of Hif1-*α* at post transcriptional level (Fig. 6A). The relative gene expression of VEGF was significantly reduced (*p* < 0.0187) in taxifolin treated cells as compared to control cells. Taxifolin caused a 0.5 fold decrease in VEGF expression at 0.125 µM and 0.6 folds decrease at 0.15 µM. A significant decrease (*p* < 0.029) in Akt levels was observed with taxifolin treatment as compared to control. Akt was downregulated by 1 fold at both treatment concentrations of taxifolin.

Immunoblotting results also depicted the reduction of VEGF, Akt and Hif1-*α* expression at protein level. In cancer cell, signal transduction begins with VEGF binding with its receptor that leads to activation of PI3K/Akt pathway. Akt activation consequently promotes activation and stability of Hif1- *α*. HepG2 and Huh7 cells treated with taxifolin showed remarkable decrease in the protein concentrations of VEGF, Akt and Hif1- *α* in dose-dependent manner. Maximum decrease in HepG2 cells was observed at 0.150 µM concentration while in Huh7 cells, maximum reduction was recorded at 0.220 µM (Fig. 7B). This effect of taxifolin is significant for the suppression of cell survival signals and the induction of apoptosis. Our results depicted attenuation in mRNA and protein expression levels of Hif1- *α*, VEGF and Akt upon taxifolin treatment in HepG2 and Huh7 cells, suggesting taxifolin can be a viable option for curbing cell survival and angiogenesis in hepatic carcinoma.

## Discussion

Naturally occurring compounds possess enhanced physicochemical properties and are demonstrated to have negative association with the risk of cancer progression. Plant based extracts have been subjected to fractionation in order to identify phytochemicals such as carotenoids, anthocyanins and polyphenols (Syed et al., 2013). According to the molecular structure, more than 4,000 varieties of flavonoids have been reported. Many of these are on the different stages of investigation being carried out on cell lines, animal models and the epidemiological trials conducted on humans (Ramos, 2008). The natural extract taxifolin is an antioxidant plant flavonoid that has been used as a liver detoxicant. It has also been observed to decrease the release of VEGF thus posing anti-angiogenic effect in cancer (Yang et al., 2003).

Evidence from previous studies show that taxifolin exhibits chemopreventive activity against colon cancer (Manigandan et al., 2015) and osteosarcoma (Chen et al., 2018). Taxifolin suppressed breast cancer by regulating the AhR and Cytochrome P450 CYP1A1 pathway (Haque & Pattanayak, 2018). Findings by Luo et al. (2018) reported that UV induced skin cancer was suppressed by taxifolin by targeting the PI3K pathway and EGFR. In view of this knowledge, we have focused on antitumor properties of a flavonoid compound taxifolin. In our study, we present the therapeutic potential of taxifolin based on in silico and in vitro-mediated regulation of angiogenic and apoptotic pathway in liver cancer. Our experiments were designed to investigate the anti-cancer activity and apoptosis induction by taxifolin on HepG2 cell line. The treatment of hepatocellular carcinoma cells HepG2 and Huh7 with taxifolin resulted in cell growth inhibition, apoptosis induction, reduction in clonogenic survival and migratory capability in a dose dependent manner. In taxifolin treated cells, a decreased expression of Hif1-*α*, VEGF, and Akt was observed. These findings are important because these three proteins serve as an important target for prevention against liver cancer.

Angiogenesis is a fundamental process in the development and progression of liver cancer. Hif1-*α* andVEGF signaling is stimulated in solid tumors resulting in cancer growth. Many diverse signaling pathways are also regulated by hypoxia-inducible factor that enables cellular response in tissue microenvironment perturbations. As Hif1-*α* is mechanistically involved in the development and progression of liver cancer, so pharmacological modifiers of Hif1-*α* in liver cancer treatment have been proposed in many studies (Ju, Colgan & Eltzschig, 2016). The high metabolic rate in tumor results in chronic hypoxia thus increasing the demand for blood supply. The same tumor cells compress the blood vessels leading to the shortage of blood supply. Hif1-*α* and VEGF are over-expressed in many tumors including liver cancer (Wen et al., 2016).

VEGF inhibition results in the blockage of angiogenesis as well as the destruction of tumor vessels. Previous in silico analysis shows that VEGF can serve as an attractive target for cancer therapy but requires in vitro validation (Lee et al., 2010). In current study, we docked taxifolin with VEGF, Akt and Hif1- *α* through AutoDockVina and found higher negative binding affinities of taxifolin with VEGF and Akt. The binding confirmation that had the highest negative binding affinity was selected to be the most favorable. The hydroxyl group (hydrogen donor) and oxygen atoms (hydrogen acceptor) contributed to the hydrogen bonding of taxifolin to the backbone (side chains) of protein residues. Docking results demonstrated that taxifolin had the highest affinity for VEGF, slightly lower for Akt and lowest for Hif1-*α*. Root Mean Square Deviation is the intermolecular distance between the atoms of superimposed proteins and is one of the important measures of molecular difference in confirmation and position (Meng, Shoichet & Kuntz, 1992). The RMSD values of ligand from crystallographically determined position were also considered according to their scoring functions. The best scores were received by proteins having the lowest RMSD values. The computational analysis by molecular docking predicted that taxifolin could alter the angiogenic and apoptotic signaling pathways. The three proteins docked against taxifolin provided evidence that taxifolin could serve as a promising agent for cancer therapy after *in vitro* validation.

Real time PCR results from previous studies suggest that taxifolin downregulated the expression of Hif1-*α* and VEGF in ovarian cancer cells OVCAR-3 and Akt in osteosarcoma U2OS cells (Luo et al., 2008). Our study demonstrated that taxifolin decreased proliferation in a dose-dependent manner by down-regulating the mRNA expression of Hif1-*α*, VEGF and Akt in Huh7 and HepG2 cells. We also demonstrated taxifolin influence on the protein levels of Hif1- *α*, VEGF and Akt in both cell lines. Immunoblotting results indicated the reduction of protein concentration of these genes in both cell lines in dose-dependent manner, revealing modulatory role of taxifolin on critical components of cancer signaling pathways i.e., Hif1-*α*,VEGF and Akt.

We further evaluated the influence of taxifolin on viability, proliferation, colony formation and migratory potential of Huh7 and HepG2 cells. Results of MTT assay showed that the percent cell viability of the taxifolin treated Huh 7 and HepG2 cells reduced in a dose-dependent manner. These results are in accordance with the previous findings in which taxifolin inhibited the growth of prostate cancer cells DU 145 at an IC50 value of 500 µM (Chen et al., 2018). Cell viability analysis revealed that taxifolin is a selective anti-cancer agent for liver cancer as it effectively induced apoptosis in HepG2 and Huh7 cell lines at an IC50 value of 0.15 µM and 0.22 µM, respectively and posed no cytotoxicity in the normal kidney cell line (Vero). This decreased proliferation observed in both cell lines treated with varying concentrations of taxifolin correlates with the growth inhibition potential of taxifolin against liver cancer.

Taxifolin treatment in human colorectal carcinoma HCT 116 cell line inhibited colonogenecity (Razak et al., 2018). Similar outcomes were obtained in our study. Taxifolin attenuated colony forming potential of Huh7 and HepG2 cell at 0.150 µM and 0.220 µM, respectively. However, in HCT116, taxifolin anti-carcinogenic affect was reported at 51.5 µM. Further, in compliance with Razak et al. taxifolin cytotoxicity increased with increased concentration. Another study reported that taxifolin inhibited the migration of human bone osteosarcoma epithelial cells U2OS (Chen et al., 2018). Cell migration assay confirmed that taxifolin selectively suppressed the migration of HCC cells but did not affect migration of the normal hepatic cells. The results of scratch assay indicate that taxifolin has a potential to inhibit wound healing in both Huh7 and HepG2 cells, therefore, can serve as a therapeutic intervention against HCC metastasis. Thus, our work presents an important approach in treating highly malignant tumor HCC.

Previously apoptosis induction (DNA fragmentation) by taxifolin was observed in Human Leukemia U937 cells by DNA ladder assay (Monasterio et al., 2004). The results of DNA ladder assay prove the induction of apoptosis indicated by DNA fragmentation in taxifolin treated HepG2 cells on agarose gel. These results provide a confirmation of the pro-apoptotic potential of taxifolin on liver cancer cell line. Furthermore, flow cytometric analysis indicated that the apoptotic cell population of HepG2 and Huh7 cells increased in number upon exposure of taxifolin. Immunoblot analysis further affirmed its pro-apoptotic role by indicating lower protein levels of PCNA, enhanced truncation of PARP and increased cleavage of caspase 3. PCNA is associated with DNA replication while PARP facilitates repairing chemotherapy induced DNA damage. Inactivation of both impairs cell’s ability to proliferate. Caspase-3 cleavage results in its activation and activated-caspase3 is integral component of both intrinsic and extrinsic apoptosis pathway (Slee, Adrain & Martin, 2001; Zhang et al., 2005). Our study highlighted the taxifolin negative modulatory control on Akt expression at transcriptional and translational level. Akt signaling brings about inhibition of intrinsic apoptotic pathway by influencing activation of Bax (Zhang et al., 2013). Hence, it can be assumed that taxifolin not only inhibits migration and angiogenesis by halting VEGF/Akt signaling cascade but also influences other Akt associated pathways that leads to induction of apoptosis.

Previous findings attribute antioxidant, chemopreventive, and pro-apoptotic characteristics to taxifolin (Brusselmans et al., 2005). Our findings further show the antineoplastic and cancer-preventive properties of the drug. The results are in accordance with the previous study demonstrating growth inhibition by taxifolin in hepatic carcinoma cells without any cytotoxic effect on healthy cells (Afsar et al., 2016; Theriault et al., 2000). We found that taxifolin did not induce cell death in normal kidney cells Vero thus validating its cytoprotective effects. This calls for further evaluation of taxifolin as a promising anticancer agent. Our experiments revealed the potential of taxifolin against liver cancer by confirming its pro-apoptotic activity in the HepG2 and Huh7 cell lines at a lower IC50 value as compared to other studies (IC50 500 µM observed by Chen et al., in prostate cancer cells DU 145) (Chen et al., 2018). Studies have found that taxifolin is a potent anti-inflammatory agent (Duan et al., 2014). In our study we found that taxifolin reduces cell viability, induces cytotoxicity, minimizes clonogenicity, reduces migration and induces DNA damage and also downregulates the expression of Hif1-*α*, VEGF, and Akt in a concentration-dependent manner.

Our results demonstrate that investigation of new methods to impede angiogenic and apoptotic signaling can considerably improve the advantages of therapeutic strategies employed in the cure of cancer. This will assist in the formulation and evolution of this compound in drug discovery and development.Taxifolin can be employed for the efficient treatment of liver cancer at molecular level and can serve as a research-based adequate contribution after further validation through in vivo and clinical trials. Thus taxifolin can be a valuable chemotherapeutic/chemopreventive agent against liver cancer in humans. Current study suggests that the pro-apoptotic potential of taxifolin against other cancers can also be investigated in future. Further in vivo studies are needed to investigate the role of taxifolin in targeting the other signaling pathways involved in cancer progression.

## Conclusions

It was revealed by the in silico docking experiments that taxifolin was able to bind with the interaction domains of Hif1-*α*, VEGF and Akt with appreciable binding energies. In summary we found that Hif1-*α*, VEGF and Akt previously overexpressed in Liver Cancer, were downregulated by taxifolin. Treatment with taxifolin inhibited the growth and viability of HepG2 and Huh7 cells with an IC50 value of 0.150 µM and 0.220 µM, respectively. In addition, our results clearly demonstrate the cytotoxic potential of taxifolin against liver cancer. Our findings also demonstrate a positive correlation of taxifolin treatment with inhibition of migration potential and with the induction of cellular apoptosis as well. Based on our results the compound taxifolin can be employed as a chemotherapeutic agent after its further validation on the animal models and humans based epidemiological studies.

##  Supplemental Information

10.7717/peerj.11276/supp-1 Supplemental Information 1 Raw Data Western blot for PCNA and AKT

10.7717/peerj.11276/supp-2 Supplemental Information 2 Raw Data Western Blot for beta actin house keep gene as loading control for VEGF and HIF 1a

10.7717/peerj.11276/supp-3 Supplemental Information 3 Raw Data Western Blot showing expression of HIF 1a and VEGF

10.7717/peerj.11276/supp-4 Supplemental Information 4 Raw Data Western blot showing expression of cleaved caspase 3

10.7717/peerj.11276/supp-5 Supplemental Information 5 Raw Data Wsetern blot showing expression of PARP and beta actin

10.7717/peerj.11276/supp-6 Supplemental Information 6 Percentage numbers of live cells, dead cells, early apoptotic and late apoptotic cells after drug treatment by flow cytometric analysis

## Additional Information and Declarations

### Data Availability

The following information was supplied regarding data availability: Raw data are available in the Supplementary Files.
