# Supplementary figures and images for: Evaluation of pro-apoptotic potential of taxifolin against liver cancer
Source: PeerJ. 2021 May 25;9:e11276. doi: 10.7717/peerj.11276 (PMC8162243; doi:10.7717/peerj.11276)

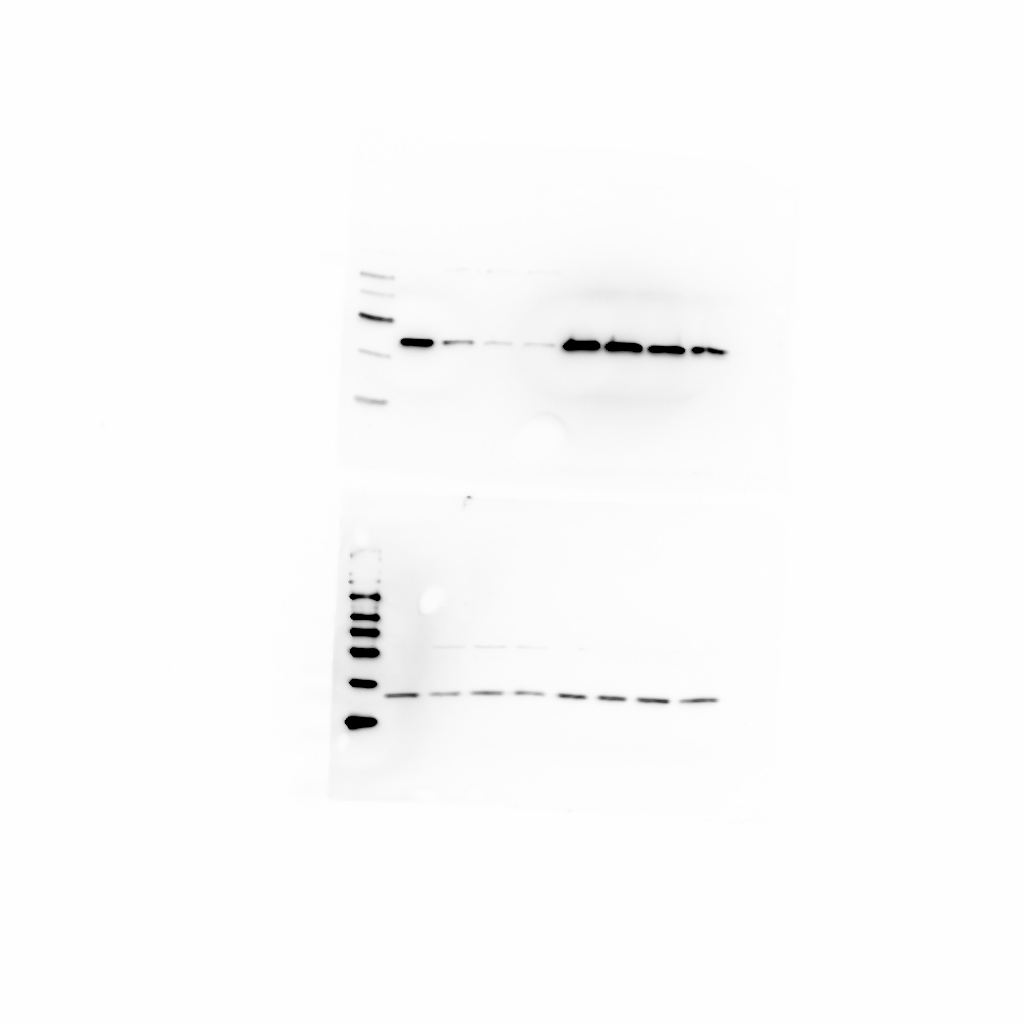

Supplement: Supplemental Information 1 — Western blot for PCNA and AKT [file peerj-09-11276-s001.jpg]

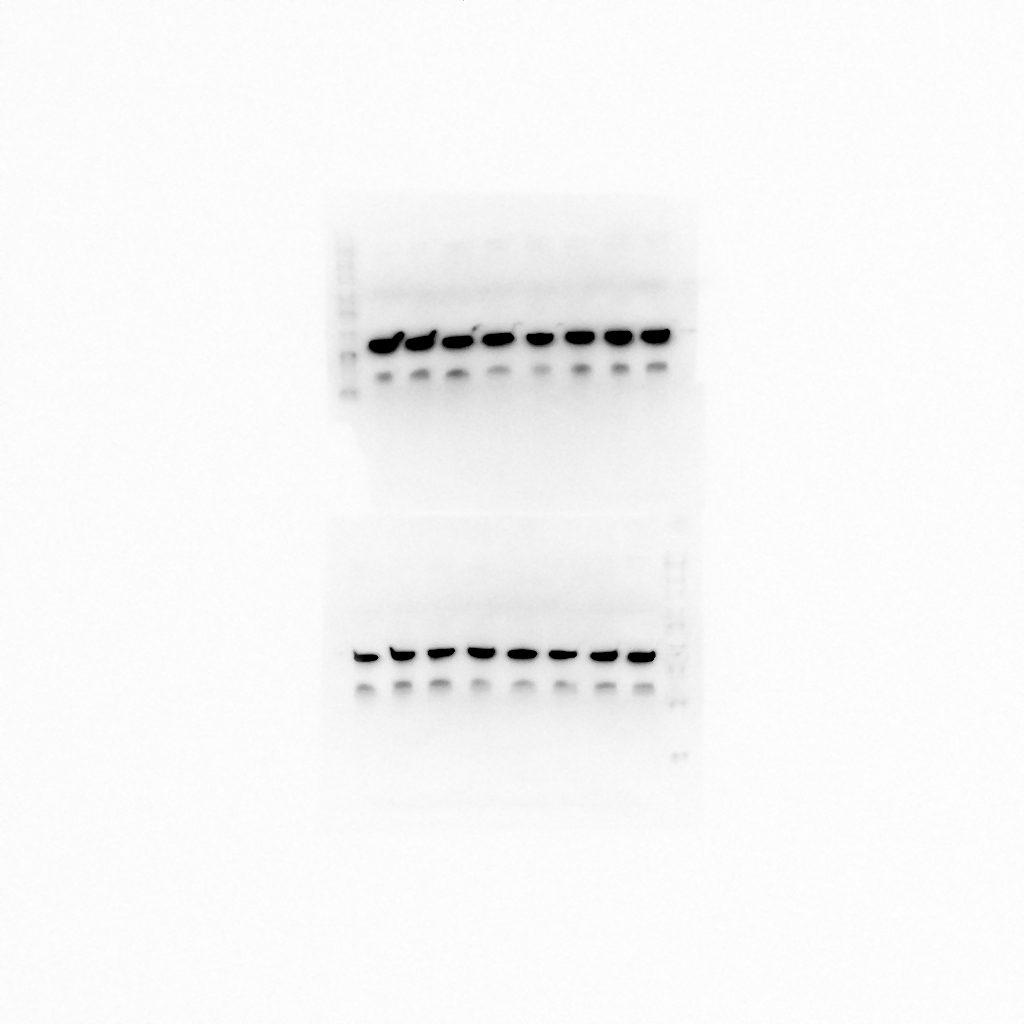

Supplement: Supplemental Information 2 — Western Blot for beta actin house keep gene as loading control for VEGF and HIF 1a [file peerj-09-11276-s002.jpg]

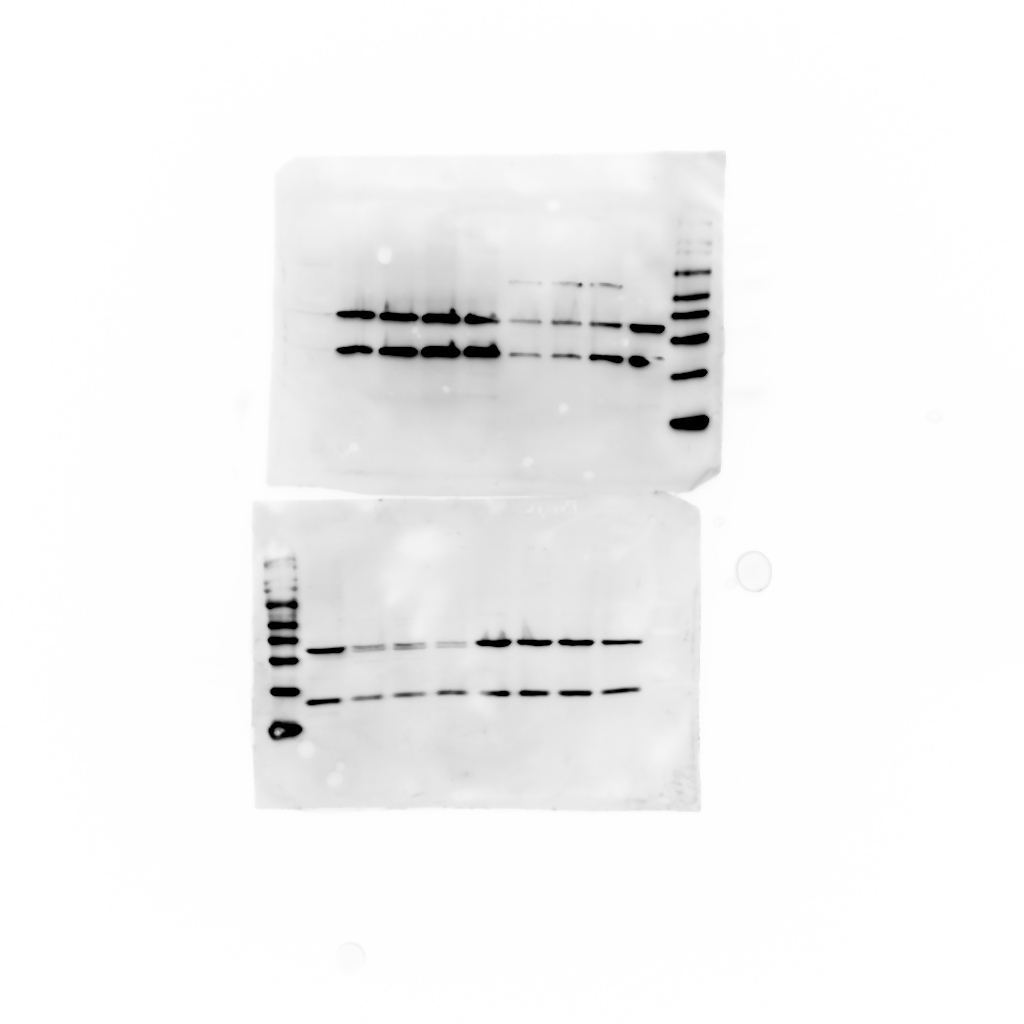

Supplement: Supplemental Information 3 — Western Blot showing expression of HIF 1a and VEGF [file peerj-09-11276-s003.jpg]

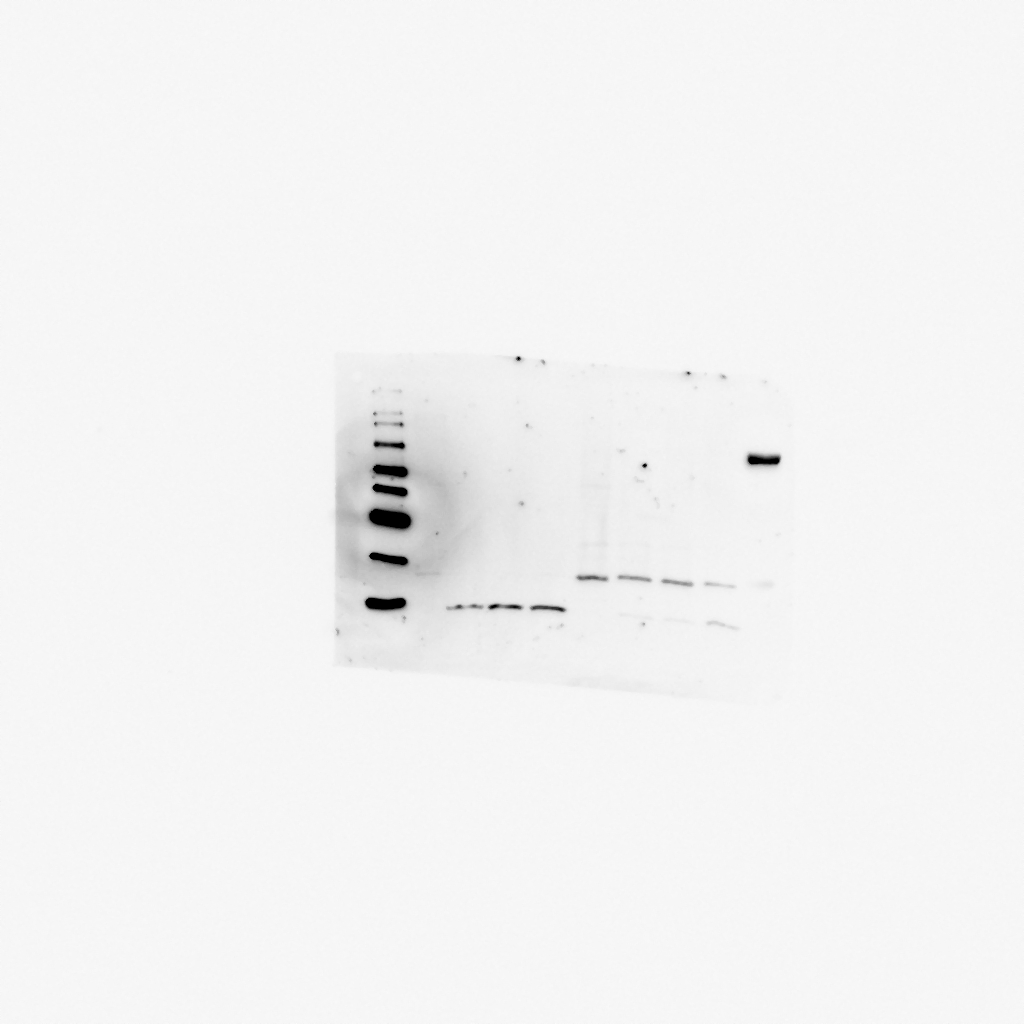

Supplement: Supplemental Information 4 — Western blot showing expression of cleaved caspase 3 [file peerj-09-11276-s004.jpg]

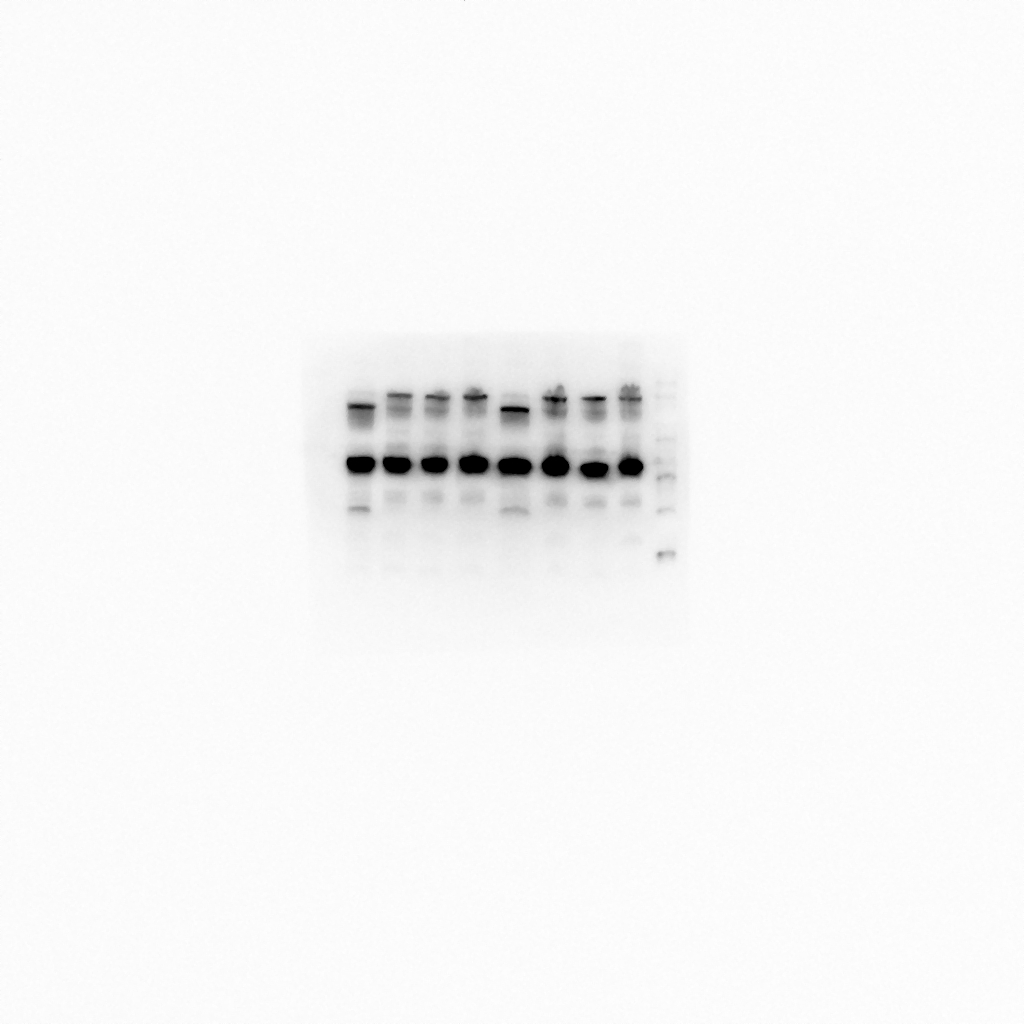

Supplement: Supplemental Information 5 — Wsetern blot showing expression of PARP and beta actin [file peerj-09-11276-s005.jpg]
