# Supplementary material for: Evaluation of pro-apoptotic potential of taxifolin against liver cancer
Source: PeerJ. 2021 May 25;9:e11276. doi: 10.7717/peerj.11276 (PMC8162243; doi:10.7717/peerj.11276)
Supplement: Supplemental Information 6 [file peerj-09-11276-s006.docx]

**Supplementary Table 1: Percentage numbers of live cells, dead cells, early apoptotic and late apoptotic cells after drug treatment and flow cytometric analysis.**

| **CELL TYPE** | **HepG2**  **Control** | **HuH 7**  **Control** | **HepG2 150nm (1)** | **HuH 7**  **150nm (2)** |
| --- | --- | --- | --- | --- |
| **Live Cells** | 94.09% | 94.17% | 11.97% | 11.23% |
| **Dead Cells** | 1.10% | 0.52% | 30.52% | 6.61% |
| **Early Apoptotic cells** | 4.57% | 0.05% | 1.41% | 6.61% |
| **Late Apoptotic cells** | 0.23% | 3.26% | 56.10% | 75.54% |
